# Plant SUMO E3 Ligases: Function, Structural Organization, and Connection With DNA

**DOI:** 10.3389/fpls.2021.652170

**Published:** 2021-04-09

**Authors:** Souleimen Jmii, Laurent Cappadocia

**Affiliations:** Department of Chemistry, Université du Québec à Montréal, Montréal, QC, Canada

**Keywords:** SUMOylation, SUMO E3 ligases, abiotic stress, structure-function analysis, DNA-binding proteins

## Abstract

Protein modification by the small ubiquitin-like modifier (SUMO) plays an important role in multiple plant processes, including growth, development, and the response to abiotic stresses. Mechanistically, SUMOylation is a sequential multi-enzymatic process where SUMO E3 ligases accelerate SUMO conjugation while also influencing target identity and interactions. This review explores the biological functions of plant SUMO E3 ligases [SAP AND MIZ1 DOMAIN-CONTAINING LIGASE (SIZs), METHYL METHANESULFONATE-SENSITIVITY PROTEIN 21 (MMS21s), and PROTEIN INHIBITOR OF ACTIVATED STAT-LIKE (PIALs)] in relation to their molecular activities and domains. We also explore the sub-cellular localization of SUMO E3 ligases and review evidence suggesting a connection between certain SUMO E3 ligases and DNA that contributes to gene expression regulation.

## Introduction

**Figure 1:**
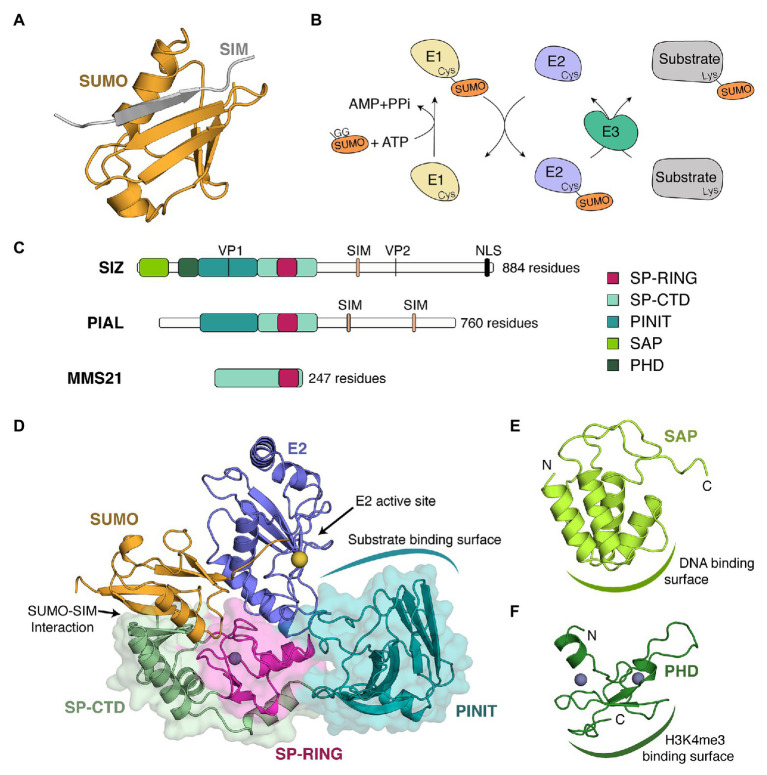
Plant SUMO E3 ligases, organizations, and structures. **(A)** Structure of a complex between SUMO and a SUMO interacting motif (SIM). In this structure, the β-sheet of SUMO (orange) from *Saccharomyces cerevisiae* is complemented by a β strand (gray) of RANBP2 (PDB 1Z5S) in an antiparallel orientation. **(B)** SUMOylation conjugation cascade where E1, E2, and E3, respectively, designate an E1-conjugating enzyme (pale yellow), an E2-conjugation enzyme (purple), and an E3-ligase (green). GG represents the di-glycine motif located at the C-terminal end of SUMO. **(C)** Schematic representation of the three types of SUMO E3 ligases found in plants. As representative members of each class, we chose *Arabidopsis thaliana* SIZ1 (top), PIAL2 (middle), and MMS21 (bottom). Domains are illustrated by boxes, whereas motifs are depicted by vertical lines. Domains present in plant SUMO E3 ligases include the SAF-A/B, Acinus, and PIAS (SAP) domain, the plant homeodomain (PHD), the PINIT domain, and the SP-RING domain. Historically, the SIM of SIZ1 has been referred to as the SXS motif (Minty et al., 2000). Although a SXS motif is well conserved in plants, this motif is actually part of a C-terminal extension of the SIM (Park et al., 2011). Studies in other systems have indeed shown that the serine residues can be targeted by phosphorylation and that this phosphorylation increases the strength of SUMO-SIM interaction by promoting interactions with a basic patch on SUMO (Chang et al., 2011; Anamika and Spyracopoulos, 2015; Cappadocia et al., 2015a). **(D)** Crystal structure of a SUMO~E2/SIZ1 complex from *S. cerevisiae* in a configuration prompt for catalysis (PDB 5JNE). The different domains of SIZ1 are in cartoon and surface representation and are colored as in **(C)**. E2 and SUMO are in cartoon representation in blue and orange, respectively. A gray sphere represents a zinc ion that stabilizes the SP-RING domain, whereas a yellow sphere represents the position of the catalytic cystine within the E2 active site. **(E)** Solution structure of the SAP domain of *Oryza sativa* (PDB 2RNO). The domain is in cartoon representation and is colored as in **(C)**. Domain termini are indicated as « N » and « C ». **(F)** Solution structure of the PHD domain of *O. sativa* (PDB 2RSD). The domain is in cartoon representation and is colored as in **(C)**. Domain termini are indicated as « N » and « C ».

SUMOylation is a reversible post-translational modification found in all eukaryotes that regulates protein activity, stability, localization as well as protein-protein interactions through their conjugation with small ubiquitin-like modifier (SUMO; Celen and Sahin, 2020). SUMOs are small proteins (10–15 kDa) that possess a conserved *β*-grasp structure composed of a five-stranded *β*-sheet that wraps around a central *α* helix (Figure 1A). They structurally resemble to Ubiquitin although their sequence similarity with Ubiquitin is limited (Cappadocia and Lima, 2018). SUMOylation contributes to numerous biological functions and it has been associated to stress responses in multiple organisms (Kurepa et al., 2003; Enserink, 2015; Seifert et al., 2015). In plants, SUMOylation is rapidly triggered by multiple stresses such as heat, drought, and salt stress (Kurepa et al., 2003; Augustine and Vierstra, 2018; Benlloch and Lois, 2018). In molecular terms, SUMOylation consists in the formation of a covalent isopeptide bond between the C-terminal end of SUMO and the lysine residue of a protein target. This conjugation is mechanistically similar to Ubiquitin conjugation and it requires the sequential activity of an E1-activating enzyme, an E2-conjugating enzyme, and E3-ligases that bring the activated E2 (E2~SUMO) in close proximity to substrates (Figure 1B; Cappadocia and Lima, 2018).

Throughout the years, two main models have emerged for protein SUMOylation. On the one hand, there is the star effect model, where the SUMOylation of a single protein results in tractable biological effects (Sarangi and Zhao, 2015). On the other hand, there is the protein group SUMOylation model, where multiple subunits of a complex are targeted to increase the cohesiveness of the complex (Psakhye and Jentsch, 2012; Jentsch and Psakhye, 2013; Augustine and Vierstra, 2018; Rytz et al., 2018). SUMOylation’s ability to regulate protein-protein interactions is linked to its capacity either to mask protein-protein interaction surfaces or to complement existing interactions with noncovalent interactions between SUMO and SUMO interacting motifs (SIM). SIMs are found in multiple SUMOylation substrates as well as in SUMO E3 ligases. They are typically composed of four hydrophobic residues forming a *β*-strand that complements the SUMO *β*-sheet in parallel or antiparallel configuration (Cappadocia and Lima, 2018; Figure 1A).

The ubiquitin pathway involves more than 1,415 E3 ligases with high-level target specificity for signaling or degradation (Craig et al., 2009). In sharp contrast, only four SUMO E3 ligases have been identified in *Arabidopsis*: SAP AND MIZ1 DOMAIN-CONTAINING LIGASE 1 (SIZ1; Miura et al., 2005), METHYL METHANESULFONATE-SENSITIVITY PROTEIN 21 (MMS21; Ishida et al., 2009), and PROTEIN INHIBITOR OF ACTIVATED STAT-LIKE 1/2 (PIAL1/2; Tomanov et al., 2014). These regulate the conjugation of at least 100 proteins (Rytz et al., 2018) and they can be divided into three general classes: SIZs, MMS21s, and PIALs (Figure 1C).

### Biological Functions of Plant SUMO E3 Ligases

#### Physiological Functions

The three types of plant SUMO E3 ligases are involved in multiple physiological processes ranging from growth regulation to stress responses (Table 1). Contrary to SUMO E1 and E2 whose deletion result in embryonic lethality (Saracco et al., 2007), the single SUMO E3 ligase knockout mutants are viable (Miura et al., 2005; Ishida et al., 2009; Tomanov et al., 2014), although they display different phenotypes. For example, *siz1* knockout plants present strong growth defects at the vegetative and reproductive stages (Ishida et al., 2009) that are caused by a strong increase in salicylic acid levels that can be rescued by the expression of NahG, a salicylate hydroxylase (Lee et al., 2007). In contrast, *mms21* knockout plants display salicylic acid-independent growth defects immediately after germination due to a decrease in cell cycle activity (Huang et al., 2009; Ishida et al., 2009). The double *siz1 mms21* mutant is embryonic lethal, thereby highlighting the important role of these two proteins in plant development (Ishida et al., 2012).

**Table 1:** Small ubiquitin-like modifier (SUMO) E3 ligase mutants in plants and their phenotype.

Type of SUMO E3 ligase	Organisms	Mutant type^1^	Plant phenotype		References
SIZs	*Oryza sativa* (OsSIZ1)	Knockout	Dwarf phenotype, reduced tiller and seed number Anther dehiscence defect and no pollen viability		Thangasamy et al., 2011
	*Arabidopsis thaliana* (AtSIZ1)	Knockout^2^	Dwarf phenotype		Lee et al., 2007; Zhang et al., 2020a
			Early flowering		Jin et al., 2008
			Secondary cell wall defect		Liu et al., 2019
			Cell expansion and proliferation defect		Catala et al., 2007
			Reduced anthocyanin accumulation		Zheng et al., 2020
			Reduced germination		Miura et al., 2009; Kim et al., 2016
			Hypersensitivity to	Excess copper	Chen et al., 2011
				Heat and drought	Miura et al., 2013; Kim et al., 2016
				Cold and freeze	Miura et al., 2007; Coleman et al., 2020
				Abscisic acid	Miura et al., 2005; Coleman et al., 2020
			Accumulation of salicylic acid		Lee et al., 2007; Miura et al., 2013
		CpSIZ1^3^ Heterologous overexpression	Delayed flowering, increased leave senescence, cold tolerance		Li et al., 2013
		OsSIZ1 Heterologous overexpression	Thermotolerance and salt tolerance		Mishra et al., 2017
	*Solanum lycopersicum* (SlSIZ1)	Overexpression	Thermotolerance		Zhang et al., 2018
	*Nicotiana tabacum*	SlSIZ1 Heterologous overexpression	Thermotolerance		Zhang et al., 2017b
	*Malus domestica* (MdSIZ1)	Overexpression	Increased rhizosphere acidification		Zhou et al., 2018
		Knockout	Downregulation of lateral root formation		Zhang et al., 2021
MMS21s	*Arabidopsis thaliana*	Knockout	Increased DNA double strand breaks Hypersensitivity to DNA damaging agents		Xu et al., 2013; Yuan et al., 2014
			Gametophyte development defect Meiosis abort and pollen tube malformation		Liu et al., 2014
			Dwarf roots phenotype, cell proliferation in the apical root meristem defect (low expression of cytokinin induced genes)		Huang et al., 2009
			Increased endoreplication		Liu et al., 2016
			Degradation of the chromatin remodeler BRAHMA in roots		Zhang et al., 2017a
			Decreased activity of the 26S Proteasome		Yu et al., 2019
PIALs	*Arabidopsis thaliana*	PIAL1 and PIAL2 double knockout	Minor disruption of carbohydrate and nitrate metabolites and downregulation of sulfur metabolism genes Salt tolerance (better PSII activity, green phenotype, and higher biomass)		Tomanov et al., 2014

1 Knockouts correspond to T-DNA insertion whereas overexpression is done using a 35S promoter.

2 Although described as a Knockout, SIZ1 mutants appear to be strong knockdowns as part of the protein is still detectable by mass spectrometry (Rytz et al., 2018).

3 CpSIZ1: *Chimonanthus praecox* SIZ1.

Concerning their biological functions, SIZ1 has been abundantly implicated in hormone signaling and the response to abiotic stress (Kim et al., 2015; Zhang et al., 2018; Coleman et al., 2020) including thermotolerance (Mishra et al., 2017; Zhang et al., 2017b, 2018). Indeed, while *siz1* knockout plants are sensitive to different stress conditions (Table 1), plants overexpressing SIZ1 are more resistant to stress (Li et al., 2013; Zhang et al., 2017b, 2018, 2020a; Mishra et al., 2017). As such, SIZ1 is now regarded as a promising candidate for crop improvement (Mishra et al., 2017). Proteomics experiments have further revealed that SIZ1 directly affects the SUMOylation of more than 100 proteins (Rytz et al., 2018) including chromatin remodeling enzymes, transcription factors, and heat-shock proteins. Whereas proteins such as HEAT SHOCK TRANSCRIPTION FACTOR A2 (HsfA2), ABSCISIC ACID INSENSITIVE 5 (ABI5) and INDUCER OF CBP EXPRESSION 1 (ICE1), three substrates of SIZ1 exhibit clear star effects (Miura et al., 2007, 2009; Cohen-Peer et al., 2010; Zhang et al., 2018), protein group modification has only been postulated for certain SIZ1 substrates. For example, multiple subunits of the SWITCH/SUCROSE NON-FERMENTABLE (SWI/SNF) complex involved in chromatin remodeling were shown to be less SUMOylated in the *siz1* mutant than in wild-type plants (Augustine and Vierstra, 2018; Rytz et al., 2018).

MMS21 has been shown to regulate DNA damage response and cell cycle regulation. Indeed, inactivation of MMS21 in *Arabidopsis* increases endoreplication by stimulating the G1/S transition while blocking G2/M (Liu et al., 2016). MMS21 facilitates the repair of genomic lesions and prevents apoptosis induced by DNA damage (Huang et al., 2009). In contrast to SIZ1, no substrates could be identified for MMS21 through proteomics approaches (Rytz et al., 2018). However, there is evidence that lack of MMS21-dependent SUMOylation on BRAHMA (BRM), an ATPase belonging to the SWI/SNF chromatin remodeling complex, leads to its degradation by the 26S proteasome (Zhang et al., 2017a). The *mms21* mutant also displays a decrease in 26S proteasome activity, which could be due to a decreased SUMOylation of REGULATORY PARTICLE TRIPLE-A ATPase subunit 2a (RPT2a), a subunit of the complex (Yu et al., 2019).

In contrast to SIZ1 and MMS21, much less is known concerning the biological roles of PIALs. These have been implicated in a salt stress response and in transcriptional silencing (Han et al., 2016) and the *pial1 pial2* knockout mutant displays improved fitness and improved photosystem II activity under stress conditions (Tomanov et al., 2014).

#### Molecular Functions

Contrary to Ubiquitin E2s that are dependent on E3s to achieve exquisite substrate specificity, SUMO E2s can directly engage substrates harboring canonical *ψ*KxE/D SUMOylation motifs (Rodriguez et al., 2001; Sampson et al., 2001; Bernier-Villamor et al., 2002). In *Arabidopsis*, about 80% of SUMOylated proteins possess a canonical SUMOylation motif (Miller et al., 2010; Rytz et al., 2018). SUMO E3 ligases contribute to SUMOylation at both canonical and non-canonical sites by performing two key roles: bringing substrates and E2~SUMO into close proximity, and stimulating SUMO discharge by the E2. Indeed, in the absence of an E3, the E2~SUMO complex is very dynamic and exhibits multiple open conformations that prevent the efficient discharge of SUMO to substrates (Pruneda et al., 2011; Page et al., 2012). By maintaining SUMO in a closed conformation with the help of SIM or SIM-like sequences (Figure 1D), SUMO E3 ligases position the thioester bond in a conformation prompt for discharge (Reverter and Lima, 2005; Cappadocia et al., 2015b; Streich and Lima, 2016).

SUMO E3 ligases can directly engage substrates through one of their protein-protein interaction domains (see below) and this results in the SUMOylation of specific proteins such as PROLIFERATING CELL NUCLEAR ANTIGEN (PCNA) in yeast (Reindle et al., 2006) and MORPHEUS MOLECULE 1 (Mom1) in plants (Han et al., 2016). SUMO can also act as a substrate for SUMO E3 ligases to promote SUMO chain formation. For example, PIAL1/2 were shown to extend SUMO chains (Tomanov et al., 2014) and are thus described either as E3 ligases (Han et al., 2016; Benlloch and Lois, 2018) or E4 ligases (Tomanov et al., 2014; Morrell and Sadanandom, 2019; Ghimire et al., 2020). Mechanistically, chain formation could be due to the presence of SIMs that contact SUMO as a substrate.

Contacting substrates through the use of protein-protein interaction domains, however, does not explain how a few SUMO E3 ligases apparently selectively modify a very large pool of substrates that share little sequence similarity between them (Rytz et al., 2018). Experiments performed in yeast suggest that the mere targeting of SUMO E3 ligases to DNA is sufficient to SUMOylate a large group of proteins in a rather promiscuous manner (Psakhye and Jentsch, 2012; Jentsch and Psakhye, 2013). Further, a proteomic study has suggested that consensus sites are not critically required for protein SUMOylation under stress conditions (Hendriks et al., 2017). It serves as an indication that the interaction of SUMO E3 ligases with their substrates might complement imperfect substrate-E2 interactions. Altogether, increasing protein SUMOylation under stress is predicted to mitigate the proteotoxic effect of stress on proteins by increasing the structural stability of proteins (Varejão et al., 2019).

### Structure of Plant SUMO E3 Domains

#### Domains Required for Activating the E2~SUMO Thioester Bond

The Siz/PIAS RING (SP-RING) domain is the most conserved domain of SUMO E3 ligases. It is composed of an *α*/*β* fold (Figure 1D) that structurally resembles the RING and U-box domains found in the Ubiquitin system (Yunus and Lima, 2009). It contains structural elements that allow interaction with the E2 and that impart specificity toward the SUMO E2 (Yunus and Lima, 2009; Streich and Lima, 2016). Mutants that alter the SP-RING of SIZ1 severely compromise SUMO conjugation (Garcia-Dominguez et al., 2008; Cheong et al., 2009).

The Siz/PIAS C-terminus domain (SP-CTD) is composed of two regions that immediately surround the SP-RING domain. In yeast SIZ1, the SP-CTD is composed of a three-stranded *β*-sheet supported by two *α*-helices (Yunus and Lima, 2009) whereas, in yeast MMS21, it is composed of *β*-hairpin-like motif packed against a *α*-helical bundle (Duan et al., 2009). Importantly, structural analysis of a SIZ1/E2~SUMO structure in yeast has revealed that the edge of a *β*-sheet of the SP-CTD domain interacts with SUMO in a SIM-like manner (Streich and Lima, 2016; Figure 1D) and maintains SUMO in a closed conformation favorable for catalysis. Comparison of the structures of SIZ1 and MMS21 reveal that the β-hairpin-like motif of MMS21 occupies the same general localization as the edge of the β-sheet in SIZ1, perhaps suggesting a similar role for these two structural elements. Whereas the SP-CTD domain is well-characterized in yeast, limited information is available in plants due to the lack of experimental investigation (i.e., structure determination or mutagenesis). Homology models, however, suggest that plant SP-CTDs could contact SUMO as their yeast counterparts.

#### Domains Required for Interacting With Other Proteins or DNA

The SAF-A/B, Acinus, and PIAS (SAP) domain is a mostly *α*-helical domain that is only present at the N-terminus of SIZ proteins. The solution structure of the SAP domain of rice SIZ1 reveals that it folds in a four-helix bundle (Suzuki et al., 2009; Figure 1E). The second and third of these helices are the most conserved regions, and they encompass a GxKxxL motif that is conserved from plants to yeast to human. This region is also the site of the DNA binding activity as assessed by NMR titration (Suzuki et al., 2009). In other organisms, the SAP domain was shown to interact with protein substrates such as RFA2 (Chung and Zhao, 2015) or p53 (Okubo et al., 2004), in addition to DNA (Psakhye and Jentsch, 2012).

The Plant HomeoDomain (PHD) is the only SUMO E3 domain that is unique to the plant kingdom. The solution structure of the PHD domain of rice SIZ1 reveals that this domain binds two zinc ions through CCHC and C4 motifs (Shindo et al., 2012; Figure 1F) and recognizes both demethylated Arg2 and trimethylated Lys4 of histone H3 (Shindo et al., 2012; Miura et al., 2020). The PHD domain is essential for the conjugation of SUMO to global transcription factor group E3 (GTE3) and it has also been suggested to contribute to the SUMOylation activity of SIZ1 (Garcia-Dominguez et al., 2008).

The PINIT domain is composed of two intertwined *β*-sheets (Figure 1D). In yeast SIZ1, this domain recognizes substrates such as PCNA (Yunus and Lima, 2009; Streich and Lima, 2016). Similarly, the PINIT domains of PIAL1/2 act as protein-protein interaction domains for the helicase MOM1 (Han et al., 2016). Although a hallmark of this domain, the eponymous PINIT motif is not perfectly conserved throughout evolution. It is PINIT in human PROTEIN INHIBITOR OF ACTIVATED STAT 1 (PIAS1), PADLT in yeast SIZ1, PIIT in *Arabidopsis* SIZ1, and PTNVT in *Arabidopsis* PIAL1/2. Mutating the PIIT motif to PAAT in *Arabidopsis* SIZ1 lowers SUMO conjugation (Cheong et al., 2009).

#### Other Motifs Found in SUMO E3 Ligases

SUMO E3 ligases contain several motifs such as SIMs, valine-proline (VP) CONSTITUTIVE PHOTOMORPHOGENESIS PROTEIN 1 (COP1) binding motifs and nuclear localization sequences (NLS; Figure 1C). SIMs are present in both SIZs and PIALs proteins. The SIM of SIZ1 is located after the SP-CTD domain, where it may facilitate interaction with a SUMO molecule tethered on the backside of the E2 (Streich and Lima, 2016). For PIAL1/2, the SIMs were shown to promote SUMO chain formation (Tomanov et al., 2014). VP motifs have only been identified in SIZ1 and they allow interaction with the substrate-binding pocket of the Ubiquitin E3 ligase COP1 (Mazur et al., 2018).

### Localization and Connection With DNA

#### SUMO E3 Ligases Are Predominantly Found in the Nucleus and Some of Them Associate With Nuclear Bodies

Cellular localization studies (Lois et al., 2003), cell fractionation studies (Saracco et al., 2007), and proteomics studies (Miller et al., 2010, 2013; Rytz et al., 2018) suggest that plant SUMOylation, similary to yeast and human SUMOylation, mostly occurs in the nucleus. The SUMOylation wave that occurs in response to stress also mostly occurs in the nucleus (Saracco et al., 2007). Importantly, the same general nuclear localization that was observed for SUMOylation is also observed for SUMO E3 ligases such as SIZ1 (Miura et al., 2005; Cheong et al., 2009; Mazur et al., 2018) and MMS21 (Ishida et al., 2009). Co-localization of SIZ1 with substrates was also shown to occur in the nucleus. Indeed, bimolecular fluorescence complementation assays indicate that the interaction between eucalyptus SIZ1 and ICE1 (Zhang et al., 2020b) and between *Arabidopsis* SIZ1 and COP1 (Lin et al., 2016) both occur in the nucleus. The exact sub-cellular localization of PIALs is unknown, although their physical and functional interaction with the nuclear protein MOM1 is consistent with a nuclear localization (Han et al., 2016; Zhao and He, 2018).

In addition to their nuclear localization, components of the SUMOylation machinery were further shown to localize to nuclear bodies in plants and in other organisms (Damme, 2010; Brown et al., 2016). Early reports demonstrated that plant SIZ1 localizes partially to nuclear punctuate structures (Miura et al., 2005; Cheong et al., 2009). Components of the SUMO machinery localize to nuclear bodies in a conjugation-dependent manner (Mazur et al., 2018), whereas SIZ1 localizes to nuclear bodies in a SP-RING-dependent manner (Cheong et al., 2009). Numerous SUMOylation substrates also localize to these nuclear bodies, including COP1, a Ubiquitin E3 ligase that regulates the stability of SIZ1 (Lin et al., 2016; Mazur et al., 2018). These studies suggest that nuclear bodies contribute to regulating the activity of SUMO E3 ligases, while also perhaps influencing their choice of substrates.

#### Co-localization of SUMO E3 Ligases With DNA and Chromatin

In mammals, the SUMO landscape on DNA is dynamic and SUMO appears to play both activating and repressing roles on gene expression (Neyret-Kahn et al., 2013). More precisely, while SUMO appears necessary for the negative regulation of many genes, it also contributes to the maximal activity of heat-stress genes (Seifert et al., 2015). This study also contributed to a model where SUMO does not work as a switch to increase or decrease transcription, but regulates the stability of protein complexes involved in gene transcription, thereby potentiating their negative or positive activity in a context-dependent manner. Heat-stress was further found to increase the association of the human SUMO E3 ligase PIAS1 to multiple genomic locations (Niskanen et al., 2015). Furthermore, the kinetics of SUMO recruitment suggests that at least part of this SUMO modification occurs directly on DNA (Seifert et al., 2015).

In plants, the genome-wide location of SUMO E3 ligases is unknown and only one study looked at the global distribution of SUMO on DNA (Han et al., 2020). The presence of SUMO on chromatin correlates with active chromatin markers, in accordance with fluorescence microscopy experiments showing that maize SUMO1 associate more with euchromatin than heterochromatin (Chen et al., 2019). Upon heat stress induction, SUMO rearranges to upregulate heat stress genes while downregulating growth genes (Han et al., 2020). Importantly, little association of SUMO to DNA occurs in the absence of SIZ1, thereby highlighting the importance of SIZ1 for targeting SUMO to DNA. Consistent with a role of SUMO as an amplifier of the stress response, the activation of stress-responsive genes and inhibition of growth-related genes were still present in plants lacking SIZ1, albeit it occurred at lower intensity than in wild-type plants (Han et al., 2020).

These studies suggest that part of the plant E3-mediated SUMOylation could occur on DNA. Indeed, plant SIZ proteins possess a SAP domain that has been shown to contact DNA (Suzuki et al., 2009). In addition, a proteomic study has shown that a good number of SIZ1 targets are transcription factors or chromatin remodeling proteins that possess DNA binding domains (Rytz et al., 2018). Using yeast two-hybrid, 76 transcription factors were also isolated as potential SUMOylation targets based on their interaction with SIZ1 or the E2 (Mazur et al., 2017). The non-sequence specific nature of SIZ1 binding to DNA even suggests that it is capable of binding near DNA-bound transcription factors, perhaps influencing lysine selection or complementing protein-protein interactions with protein-DNA interactions. More than just contacting DNA, SIZ1 could interact with open chromatin through its PHD domain that was shown to interact with tri-methylated histone H3K4 (Shindo et al., 2012; Miura et al., 2020). Also, SIZ1 was recently shown to bind and increase the SUMOylation of the DNA demethylase REPRESSOR OF SILENCING 1 (ROS1), thereby increasing its stability and activity and altering the methylation pattern in thousand genomic locations (Kong et al., 2020). Whether this interaction occurs directly on DNA is, however, still unknown. Finally, there is evidence that DNA binding affects the activity of SUMO E3 ligases as, in yeast, MMS21 SUMOylation activity is stimulated by the binding of the complex MMS21/STRUCTURAL MAINTENANCE OF CHROMOSOME 5 (SMC5)/STRUCTURAL MAINTENANCE OF CHROMOSOME 6 (SMC6) to DNA (Varejão et al., 2018).

### Conclusion and Perspectives

SUMO E3 ligases facilitate the SUMOylation of multiple proteins, particularly under stress conditions. This requires a complex interplay between the different domains and motifs of plant E3 ligases to achieve optimal subcellular targeting, contact relevant substrates, and stimulate catalysis by the E2~SUMO complex. Recent evidence now suggests that DNA targeting by SUMO E3 has a profound influence on activity and the choice of substrates. Outstanding questions for the future include: (i) Does DNA- or chromatin-binding by SUMO E3 ligases modify their SUMO E3 activity? (ii) Does DNA- or chromatin-binding of transcription factors or chromatin modulators modify their susceptibility to SUMOylation or the choice of target lysine residues? (iii) Does stress promote the association of plant SUMO E3 ligases to DNA? (iv) How are SUMO E3 ligases distributed on DNA during normal growing conditions and under stress? Finally, while this review focused on the role of SUMO E3 ligases on transcription, we also expect that future studies will highlight the role of plant SUMO E3 ligases and DNA-targeting on DNA damage response.

## Author Contributions

SJ and LC drafted and edited the manuscript. SJ made Figure 1; Table 1. Both the authors contributed to the article and approved the submitted version.

### Conflict of Interest

The authors declare that the research was conducted in the absence of any commercial or financial relationships that could be construed as a potential conflict of interest.
